# Demoralization and dignity loss in breast cancer: A network analysis and computer simulation study

**DOI:** 10.1016/j.apjon.2025.100803

**Published:** 2025-10-11

**Authors:** Ying Xiong, Hongman Li, Keqing Cai, Miao Yu, Jian Zhou, Jiaying Li, M. Tish Knobf, Zengjie Ye

**Affiliations:** a School of Nursing, Guangzhou University of Chinese Medicine, Guangzhou, Guangdong Province, China; b The First School of Clinical Medicine of Guangzhou University of Chinese Medicine, Guangzhou, Guangdong Province, China; c The First Affiliated Hospital of Guangzhou University of Chinese Medicine, Guangzhou, Guangdong Province, China; d The Nethersole School of Nursing, Faculty of Medicine, The Chinese University of Hong Kong, Hong Kong Special Administrative Region of China; e School of Nursing, Yale University, Orange, CT, United States; f School of Nursing, Guangzhou Medical University, Guangzhou, Guangdong Province, China

**Keywords:** Demoralization, Dignity, Interrelationship analysis, Bayesian network analysis, Computer-simulated intervention

## Abstract

**Objective:**

Demoralization and loss of dignity frequently co-occur among individuals with cancer. However, their symptom-level associations remain poorly characterized. This study aimed to delineate the underlying pathways linking demoralization and dignity loss in breast cancer survivors and to identify potential symptom targets for intervention.

**Methods:**

A total of 411 female breast cancer survivors were assessed using the Demoralization Scale II and the Patient Dignity Inventory. A Gaussian graphical model was used to identify central and bridge symptoms, while a Bayesian network model estimated the directional associations and potential causal pathways between demoralization and dignity-related symptoms. Computer-simulated intervention analyses were conducted to determine which symptom reduction would yield the greatest decrease in overall network activation.

**Results:**

In the Gaussian network, illness uncertainty emerged as the most central symptom (strength ​= ​1.20), and loss of life meaning as the principal bridge symptom (bridge strength ​= ​0.30). The Bayesian network identified distress as an upstream node triggering downstream cascades from demoralization to dignity loss. Simulation analyses indicated that reducing distress led to the largest decrease in global network activation (from 5.3% to 1.9%).

**Conclusions:**

This study elucidates the symptom-level mechanisms through which demoralization contributes to dignity loss in breast cancer survivors, highlighting loss of life meaning as a key bridging factor. Targeting distress may represent an effective intervention strategy to concurrently alleviate demoralization and preserve dignity.

## Introduction

### Background and aims

Breast cancer remains a leading global health concern, ^1^ with China reporting 357,200 new cases in 2022 alone.^2^ Advances in adjuvant therapy have improved survival,^3^ but survivors often face enduring physical sequelae and heightened psychological distress. Patients undergoing chemotherapy face both physical and psychological suffering. The physical impairments caused by chemotherapy, such as hair loss,^4^ nausea and vomiting,^5^ are intertwined with the illness uncertainty brought by a cancer diagnosis.^6^ These direct physical impairments lead to a loss of dignity.^7^ At the same time, the constant fear of cancer recurrence during chemotherapy undermines patients' emotional functioning.^8^ It triggers a crisis of meaning and a deep sense of helplessness, which is one of the core characteristics of demoralization.^9^ Hence, demoralization and loss of dignity are especially pervasive in this population, profoundly shaping survivors’ adjustment and long-term well-being and underscoring the urgent need for dedicated psychosocial support.

Demoralization, characterized by pervasive hopelessness, perceived incompetence, and existential disorientation,^10^ affects 13.5% to 49.4% of cancer patients^11^ with even 28.1% of breast cancer cohorts exhibiting a high level of demoralization.^12^ In breast cancer patients, demoralization manifests through symptoms such as dysphoria, disheartenment, and helplessness.^9^ These demoralization symptoms predict suicidal ideation and behavior.^13^ By eroding coping resources and undermining life meaning, demoralization imposes a substantial mental health burden on patients with breast cancer. Dignity, defined as preservation of self-respect, self-esteem,^14^ and personal values, is likewise vulnerable in cancer patients.^15^ Up to 46% of patients report dignity-related distress.^16^ Among breast cancer survivors, loss of dignity manifests primarily as dissatisfaction with the loss of femininity and changes in body image, as well as discomfort from reduced social roles.^17^^,^^18^ Loss of dignity correlates with poorer quality of life and increased depression,^19^^,^^20^ underscoring the critical importance of maintaining dignity in breast cancer care.

Previous studies have explored the association between demoralization and dignity loss,^20^, ^21^, ^22^, ^23^ discovering that demoralization is positively correlated with dignity loss and that dignity loss mediates the relationship between demoralization and quality of life.^20^ This suggests that demoralization influences dignity loss. However, another study points out that dignity loss mediates 81% of the effect of the number of physical problems on demoralization,^21^ indicating that dignity loss influences demoralization. It is evident that while the two variables are correlated,^20^^,^^21^ the directional and precise nature of their interrelationship remains controversial. Additionally, relevant studies have been macro-level investigations at the total scores, whereas the micro-level connections between the two at the symptom level remain unclear. Clarifying the associative pathways and directional links between demoralization and dignity loss is therefore essential for precision psychosocial care, as it may help inform whether interventions should primarily target demoralization to preserve dignity, or conversely, protect dignity to mitigate demoralization. Moreover, moving beyond macro-level total scores to examine symptom-level associations can uncover specific pathways through which particular aspects of demoralization interact with components of dignity loss. Such micro-level insights can identify critical nodes within this interplay, offering a more nuanced understanding of patient experiences and enabling the development of targeted, symptom-focused interventions. In doing so, research can not only refine theoretical models but also provide more precise and effective strategies to improve patients’ quality of life.

Network analysis systematically maps complex symptom interdependencies and pinpoints both central and bridge nodes for targeted intervention.^24^ Central nodes refer to the most influential symptoms for overall network^25^ while bridge nodes represent the most connecting symptoms between different communities.^26^ Bayesian network analysis can then model directional relationships and estimate probabilistic dependencies between demoralization and dignity loss,^27^ advancing our understanding of their dynamic interactions in clinical populations. Finally, computer-simulated interventions, an innovative alternative to traditional clinical trials, allow in simulated manipulation of individual symptoms to evaluate their effects on the overall network,^28^ thereby guiding development of dual-action, symptom-focused treatments. This approach is implemented through computer algorithms, prioritizing the identification of highly effective intervention targets without the need for costly clinical trials.^28^ It reduces the costs of trial and error and avoids the waste of resources. Psychological phenomena often manifest as nonlinear interactions between symptoms, such as vicious cycles. A network perspective is more suitable for modeling such complexity, and computer simulation is a natural extension of intervention research in testing demoralization and dignity loss. Additionally, simulation can validate network analysis results, such as whether “the higher the symptom centrality, the stronger the intervention effect,” thereby accelerating translational application of further interventions for demoralization and dignity loss.

In this study, we aim to (1) identify central and bridge symptoms within or linking demoralization and dignity loss; (2) model the probabilistic dependencies and directional associations between these symptoms; and (3) simulate symptom-level interventions to pinpoint optimal dual-action targets.

### Theoretical framework

Proposed by Chochinov,^29^ the dignity model provides a comprehensive conceptual basis for understanding the multidimensional factors that influence a patient's perceived dignity, particularly in the context of severe illness and cancer survivorship. Structurally, the model comprises three interrelated domains: (1) the dignity-conserving repertoire, encompassing continuity of self, preservation of social and functional roles, maintenance of pride, autonomy, and hope; (2) the social dignity inventory, which reflects interpersonal dynamics, privacy boundaries, perceived burden to others, and the quality of social support; and (3) illness-related concerns, such as symptom burden, functional impairment, and physical decline.^30^ Within this framework, dignity is conceptualized as a product of dynamic interactions among psychological, physical, and social variables. Predisposing factors interact with precipitating factors to erode dignity over time.

According to the dignity model,^29^ demoralization may precipitate dignity loss via three mechanisms. First, cognitive resource depletion, where pervasive hopelessness undermines autonomy and self-worth, leads to core dignity collapse.^11^ Second, social feedback reinforcement, through withdrawal that reduces support and amplifies negative interpersonal cues, accelerates erosion of social dignity.^31^ Third, body–dignity interaction, where somatic symptoms such as pain heighten helplessness and self-worth denial, completes a cascade from internalized demoralization to externalized dignity distress.^32^

We therefore hypothesize that demoralization symptoms act as antecedent drivers, triggering subsequent dignity-related symptoms in breast cancer survivors.

## Methods

### Participants

The Be Resilient to Breast Cancer (BRBC) project enrolled 420 women with histologically confirmed breast cancer from five tertiary hospitals in Guangdong, Hunan, and Sichuan provinces between July and October 2024.^33^, ^34^, ^35^, ^36^ The data was collected through face-to-face paper questionnaires filled by patients themselves. After excluding nine participants with incomplete data, 411 (97.9% response) were retained. Inclusion criteria were: (1) Women aged ≥ 18 years diagnosed with breast cancer based on NCCN guidelines;^37^ (2) Currently receiving adjuvant or neoadjuvant chemotherapy regimens based on NCCN guidelines;^37^ (3) Able to communicate effectively in Mandarin; and (4) Signed a written informed consent form. Exclusion criteria were as follows: (1) Severe hearing or visual impairments, as determined by the investigator during the initial interaction, that prevent understanding of the study questions or providing informed consent; (2) Confirmed dementia or cognitive impairment in clinical history; (3) A previous history of major mental illness.

### Sample size

A priori sample size calculation was not performed for this specific network analysis, as the data were obtained from the larger Be Resilient to Breast Cancer (BRBC) project. Nevertheless, the adequacy of the obtained sample (*n* ​= ​411) for providing stable network estimates was evaluated using post-hoc stability analyses, the results of which are presented in the following results section.

### Variables and their assessment

Demoralization was assessed with the 16-item Chinese version of the Demoralization Scale II (DS-II), adapted from Robinson et al.^38^ and translated by Wu et al.^39^ Respondents rate each statement from 0 (“never”) to 2 (“often”) on a 3-level Likert scale, producing a total score of 0–32, with higher scores denoting greater demoralization. In our sample, the DS-II demonstrated excellent internal consistency (Cronbach's α 0.90). Detailed item content and scoring are provided in Supplementary Table S1.

Dignity was measured using the 25-item Patient Dignity Inventory (PDI), originally developed by Chochinov et al.^31^ and culturally adapted by Ge et al.^40^ Participants rate each item from 0 to 4 on a 5-level Likert scale, yielding higher scores for more severe dignity impairment. The PDI showed high reliability in this cohort (Cronbach's α 0.92), and full item descriptions appear in Supplementary Table S2. Both variables were considered as continuous variables in this study.

### Data analysis

We first estimated an undirected Gaussian graphical model to characterize symptom-level associations between demoralization and dignity loss. The node groups were defined based on the source questionnaires. The demoralization group consisted of all 16 items from the DS-II, and the dignity loss group consisted of all 25 items from the PDI. Node strength and bridge strength were calculated to determine the core and bridge symptoms respectively.^41^^,^^42^ Z-score conversion was performed to standardize the values of node strength and bridge strength for better graphing. The top three nodes with the highest strength were identified as core symptoms. Bridge nodes were selected through an 80th percentile cutoff on bridge strength scores^26^ and were highlighted in red in the final bridge network visualization. Graphical Lasso was used to select the optimal regularization parameter, thereby retaining edges with strong correlations to simplify the network. Within the bridge network, green edges represent positive associations and white rings encircling nodes signify the proportion predicted by adjacent nodes. Edge-weight accuracy was assessed via 95% bootstrap confidence intervals (CIs), and network stability was evaluated using the correlation stability coefficient (CS-coefficient), with scores over 0.25 being acceptable and over 0.50 being optimal.

We then constructed a directed acyclic graph using a Gaussian Bayesian network with the hill-climbing algorithm to model potential directional pathways among symptoms.^27^ We assessed network robustness and edge-direction stability over 1000 bootstrap resamples, and only edges whose directionality consistently exceeded the predefined threshold were retained in the final Bayesian network.^43^^,^^44^ Edge weight or edge strength refers to the strength of the conditional probability relationship between symptoms, reflected by the thickness of the edge. The higher the absolute value of the edge weight, the thicker the corresponding edge, indicating a stronger association.^27^

Finally, to simulate symptom-targeted interventions, item scores were dichotomised (0 vs. ≥ 1) and an Ising model was fitted. The NodeIdentifyR algorithm generates simulated data by systematically adjusting the threshold parameters of symptoms and compares the changes in network symptoms before and after intervention. It outputs the symptom with the largest change in total score after intervention as the optimal target for intervention.^28^ This algorithm then identified two kinds of targets (1) alleviation targets, whose simulated weakening maximally reduced overall network connectivity; and (2) aggravation targets, whose simulated strengthening maximally increased connectivity.^28^

All analyses were conducted in R version 4.4.1 using the packages “ggplot2”, “qgraph”, “bootnet”, “networktools”, “bnlearn”, and “NodeIdentifyR”.

## Results

### Participants’ demographics and characteristics

Among 411 female breast cancer survivors, mean age was 50.8 years (standard deviation [SD]: 11.2; range: 28–85). Most were married (87.1%) and urban dwellers (73.5%). And 48.4% participants had attained primary education or below. The luminal B subtype accounted for 45.7% (*n* ​= ​188) and stage II disease for 43.3% (*n* ​= ​178) (Table 1).

**Table 1:** Demographic characteristics of patients (*N* = 411).

Variables	*n* (%)
**Age (M** ± **SD)**	50.8 ​± ​11.2
**Bodymassindex (M** ± **SD)**	23.1 ​± ​3.7
**Marriage**	
Unmarried	12 (2.9%)
Married	358 (87.1%)
Divorced/widowed	41 (10.0%)
**Fertility status**	
Childless	31 (7.5%)
One child or more	380 (92.5%)
**Menopause**	
Yes	185 (45.0%)
No	226 (55.0%)
**Residence**	
Urban area	302 (73.5%)
Rural area	109 (26.5%)
**Medical insurance**	
Medical insurance for employees	231 (56.2%)
Medical insurance for residents	159 (38.7%)
Others	21 (5.1%)
**Financial burden**	
No burden	74 (18.0%)
Mild-moderate	185 (45.0%)
Heavy	152 (37.0%)
**Educational background**	
Primary school and below	199 (48.4%)
High school	87 (21.2%)
Undergraduate	120 (29.2%)
Postgraduate and above	5 (1.2%)
**Occupation status**	
Employed	131 (31.9%)
Unemployed	124 (30.2%)
Retired	156 (38.0%)
**Stage of disease**	
I	121 (29.4%)
II	178 (43.3%)
III	64 (15.6%)
IV	48 (11.7%)
**Pathological type**	
Non-invasive carcinoma	45 (10.9%)
Invasive carcinoma	366 (89.1%)
**Molecular subtype**	
Luminal A	61 (14.8%)
Luminal B	188 (45.7%)
HER-2 overexpression	102 (24.8%)
Triple negative	60 (14.6%)
**Surgery**	
Unoperated	68 (16.5%)
Simple mastectomy	126 (30.7%)
Breast-conserving therapy	94 (22.9%)
Radical mastectomy	123 (29.9%)
**Family history**	
Yes	61 (14.8%)
No	350 (85.2%)

M, mean; SD, standard deviation.

### Undirected network analysis

**Fig. 1:**
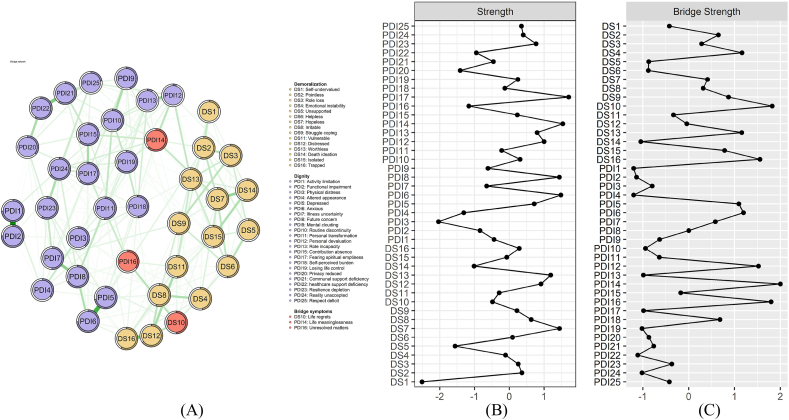
Undirected network between demoralization and dignity loss. (A) bridge network structure; (B) centrality indices; (C) bridge centrality indices.

The final bridge network (Fig. 1A) identified “illness uncertainty” (PDI 17; strength ​= ​1.200), “life meaninglessness” (PDI 14; strength ​= ​1.172), and “anxious” (PDI 6; strength ​= ​1.164) as the most central symptoms (Fig. 1B). Key bridge symptoms were “life meaninglessness” (PDI 14; bridge strength ​= ​0.299), “life regrets” (DS 10; bridge strength ​= ​0.282), and “unresolved matters” (PDI 16; bridge strength ​= ​0.280) (Fig. 1C). All edges presented in this network were green, indicating the positive correlations between all these symptoms. Bootstrap 95% CIs for edge weights indicated high accuracy (Supplementary Fig. S1). And a case-dropping bootstrap yielded the same CS-coefficient of 0.52 for strength, closeness, bridge strength and bridge expected influence, except for 0.25 for betweenness, confirming network stability (Supplementary Fig. S2).

### Gaussian Bayesian network analysis

**Fig. 2:**
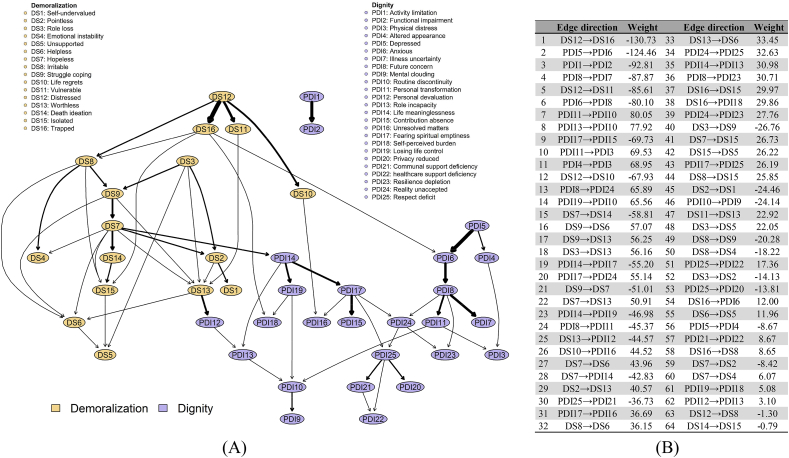
Bayesian network between demoralization and depression. (A) Bayesian network structure; (B) edge direction and weight in the Bayesian network. The higher edges indicate the higher correlation magnitude across symptoms.

The directed acyclic graph (Fig. 2A) positioned “Distressed” (DS12) as the primary parent node, directly influencing “trapped” (DS16), “vulnerable” (DS11), “life regrets” (DS10), and “irritable” (DS8), with edge directions and weights are shown in Fig. 2B. Most dignity nodes lay downstream of demoralization symptoms, suggesting a directional cascade. Notable pathways included “distressed→trapped→anxious” (DS12→DS16→PDI 6), “distressed→life regrets→unresolved matters” (DS12→DS10→PDI 16), and “role loss→struggle coping→hopeless→life meaninglessness” (DS3→DS9→DS7→PDI 14), corroborating the centrality of “anxious” (PDI 6), “unresolved matters” (PDI 16) and “life meaninglessness” (PDI 14). The positioning of “Distressed” as the primary parent node suggests that the subjective experience of pervasive distress may be a foundational trigger, initiating a cascade of other symptoms. In practical terms, this means interventions targeting early nodes like distress may have a broader downstream effect on preserving dignity and alleviating demoralization. Additionally, these pathways represent actionable clinical hypotheses. They pinpoint that interventions focusing on key symptoms in directional chains such as “anxious”, “unresolved matters” and “life meaninglessness” might break the transmission of symptom chains and prevent the progression to dignity loss.

### Computer-simulated intervention

**Fig. 3:**
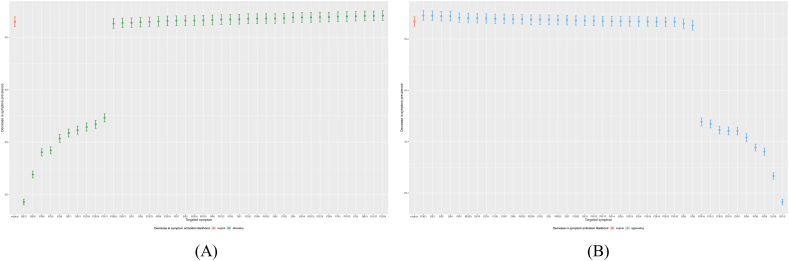
Simulated intervention targets in the symptom network. (A) alleviating symptoms; (B) aggravating symptoms.

Computer-simulated intervention (Fig. 3) confirmed that weakening “distressed” (DS12) yielded the greatest reduction in overall network activation, from 5.3% to 1.9% (Fig. 3A). Conversely, strengthening “communal support deficiency” (PDI 21) minimally increased activation, from 5.3% to 5.5% (Fig. 3B). Further simulation outputs are provided in Supplementary Fig. S3. The results indicate that interventions targeting “distressed” are most effective in suppressing symptom activation across the entire network, while the impact of “communal support deficiency” is relatively limited. This provides key computational evidence for prioritizing intervention targets. More information can be found in Supplementary Fig. S3.

### Post-hoc network stability analysis

To evaluate the reliability of our network model with *n* ​= ​411 participants, we conducted a post-hoc network stability analysis.^45^ The correlation stability coefficient (CS-coefficient) for strength and bridge strength was 0.52, which exceeds the recommended threshold of 0.5, confirming reliable estimation of network parameters with 411 participants.

## Discussion

### Main findings

This study is, to our knowledge, the first to map symptom-level associations and model possible directional pathways between demoralization and dignity loss in breast cancer survivors, and to identify optimal dual-action intervention targets. Our findings demonstrate that demoralization symptoms may precipitate dignity impairment via multiple symptom cascades and that alleviating “distressed” yields the greatest reduction in overall symptom burden, offering a precise focus for psychosocial care. Clinically, our findings enable therapists to prioritize therapies that reduce this core symptom for maximal downstream benefits. This supports a more efficient, mechanism-focused approach to palliative care rather than a broad-based one. Theoretically, mapping the symptom networks provides a practical model for clinicians to understand and explain how demoralization evolves into dignity loss for individual patients. This lays a solid theoretical foundation for constructing precise intervention measures of demoralization and dignity loss in future clinical practice.

In the undirected network, “illness uncertainty,” “life meaninglessness,” and “anxious” emerged as the most central symptoms, while “life meaninglessness,” “life regrets,” and “unresolved matters” served as key bridges. The dual role of “life meaninglessness” as both a central and a bridging symptom highlights its unique position at the intersection of demoralization and dignity loss. This indicates that it not only activates the entire network and triggers other symptoms, but also acts as the main channel through which demoralization erodes dignity. Therefore, alleviating the “life meaninglessness” is expected to produce a dual therapeutic effect: it can both reduce the severity of the whole symptoms and cut off the main pathway through which demoralization damages dignity. This suggests that interventions such as meaning-centered therapy may simultaneously attenuate demoralization and preserve dignity.^46^

The Bayesian network further demonstrated potential directional relationships between symptoms. “Distressed” functioned as the primary parent node, driving downstream symptoms including “trapped,” “vulnerable,” “life regrets,” and “irritable.” Most dignity-related symptoms lay downstream of this demoralization core, supporting our hypothesis that demoralization may trigger subsequent dignity loss. Notably, key directed chains included “distressed→trapped→anxious,” “distressed→life regrets→unresolved matters,” and “role loss→struggle coping→hopeless→life meaninglessness.” Additionally, the nodes “anxious,” “unresolved matters,” and “life meaninglessness,” which were previously identified as central or bridge symptoms in the undirected analysis, also serve as pivotal mediators in these causal pathways. This underscores their dominant influence in the demoralization-dignity network and their critical role in the emergence of dignity impairment. In clinical practice, these findings provide a precise framework for targeting interventions. By recognizing “anxious” as a pivotal mediator, clinicians can implement cognitive-behavioral strategies such as exposure therapy and cognitive restructuring to reduce avoidance behaviors and catastrophic thinking.^47^ For unresolved matters, narrative therapy^48^ can help patients reorganize fragmented life stories and find closure through experience-sharing activities. Moreover, for life meaninglessness, clinicians could employ meaning-centered therapy^46^ to assist patients in exploring values, relationships, and achievements, thereby effectively disrupting the cascade from demoralization to dignity loss and thereby safeguarding patient dignity.

Computer-simulated intervention identified “distressed” as the most effective alleviation target and “communal support deficiency” as the principal aggravator, although the latter produced only a marginal increase in network activation. This simulation-based approach provides critical validation for our theoretical model, demonstrating that computational methods can effectively identify high-value intervention targets in psychopathological networks, despite disparities in simulated intervention effects. Hence, “distressed” should be considered as an optimal targeted symptom. Targeting “distressed”, the primary parent node, yielded the greatest reduction in overall symptom connectivity, effectively interrupting the cascade from demoralization to dignity loss. These findings highlight distress modulation as a pragmatic intervention focus that can be translated into stepped-care clinical strategies.

### Implications for nursing practice and research

In personalized care, caregivers could prioritize assessing patients' levels of “distressed” symptoms and then select appropriate cognitive-behavioral therapy^49^ based on severity. For mild cases, psychological education may be used; for moderate cases, cognitive restructuring may be used; and for severe cases, more in-depth behavioral experiments and exposure therapy may be required. Thereby, both demoralization and dignity impairment could be mitigated simultaneously.

### Limitations

This study has several limitations. First, the sample, drawn from tertiary centers in three Chinese provinces, may not represent the broader breast cancer population. Second, our network analysis did not adjust for potential confounders, such as age, disease stage, or comorbid psychological symptoms. The observed associations between demoralization and dignity loss could therefore be subject to confounding bias, and the potential directional relationships discussed should be interpreted with caution. Third, dichotomizing symptom scores for the Ising model may have obscured severity gradients, underscoring the need for network methods that retain continuous or ordinal information. Finally, the cross-sectional design limits the ability to draw firm conclusions regarding causal inference. Although the model estimates possible associative pathways, these remain speculative rather than confirmatory in the absence of longitudinal validation. Future studies incorporating repeated-measures or panel data are needed to evaluate the persistence of these network relations and to better support inferences about their directionality over time.

## Conclusions

Demoralization appears to precipitate dignity loss through specific symptom cascades, notably those centered on life meaninglessness and distress. Targeting distress offers the greatest potential to disrupt this cascade, yielding concurrent reductions in both demoralization and dignity impairment. Incorporating distress-focused interventions into psychosocial care may therefore optimize outcomes for breast cancer survivors.

## Declaration of competing of interest

The authors declare no conflict of interest. The corresponding author, Prof. Zengjie Ye, is an editorial board member of *Asia–Pacific Journal of Oncology Nursing*. The article was subject to the journal's standard procedures, with peer review handled independently of Prof. Ye and their research groups.

## CRediT authorship contribution statement

**Ying Xiong**: Formal analysis, Writing – original draft. **Hongman Li**: Conceptualization, Data curation. **Keqing Cai**: Investigation, Software. **Miao Yu**: Investigation, Methodology. **Jian Zhou**, **Jiaying Li**, **M.** **Tish Knobf** and **Zengjie Ye**: Supervision, Writing – review & editing. All authors read and approved the final manuscript.

## Ethics statement

This study was approved by the Ethics Committee of the First Affiliated Hospital of Guangzhou University of Traditional Chinese Medicine (Approval No. K-2024-202) and was conducted in accordance with the 1964 Helsinki Declaration and its later amendments or comparable ethical standards. All participants provided written informed consent.

## Data availability statement

The data that support the findings of this study are available from the corresponding author, ZY, upon reasonable request.

## Declaration of generative AI and AI-assisted technologies in the writing process

No AI tools were used during the preparation of this work.

## Funding

This research was funded by grants from the National Natural Science Foundation of China (No. 72274043, 71904033), the Young Elite Scientists Sponsorship Program by CACM (No. 2021-QNRC2-B08), Guangdong Philosophy and Social Science Foundation (No. GD25YSH17), and Sanming Project of Medicine in Shenzhen (No. SZZYSM202206014). The funders had no role in considering the study design or in the collection, analysis, interpretation of data, writing of the report, or decision to submit the article for publication.
