# Supplementary material for: Demoralization and dignity loss in breast cancer: A network analysis and computer simulation study
Source: Asia Pac J Oncol Nurs. 2025 Oct 11;12:100803. doi: 10.1016/j.apjon.2025.100803 (PMC12595355; doi:10.1016/j.apjon.2025.100803)
Supplement: Multimedia component 1 [file mmc1.docx]

**Supplementary Materials**

|  | Supplementary Table S1: Items for Demoralization Scale-II (DS-II). | |
| --- | --- | --- |
| Dimensions | Node names | Node numbers and items |
| Meaning and purpose | Self-undervalued | DS1. There is little value in what I can offer others. |
| Meaning and purpose | Pointless | DS2. My life seems to be pointless. |
| Meaning and purpose | Role loss | DS3. My role in life has been lost. |
| Stress and coping | Emotional instability | DS4. I no longer feel emotionally in control. |
| Meaning and purpose | Unsupported | DS5. No one can help me. |
| Meaning and purpose | Helpless | DS6. I feel that I cannot help myself. |
| Meaning and purpose | Hopeless | DS7. I feel hopeless. |
| Stress and coping | Irritable | DS8. I feel irritable. |
| Stress and coping | Struggle coping | DS9. I do not cope well with life. |
| Stress and coping | Life regrets | DS10. I have a lot of regret about my life. |
| Stress and coping | Vulnerable | DS11. I tend to feel hurt easily. |
| Stress and coping | Distressed | DS12. I feel distressed about what is happening to me. |
| Meaning and purpose | Worthless | DS13. I am not a worthwhile person. |
| Meaning and purpose | Death ideation | DS14. I would rather not be alive. |
| Stress and coping | Isolated | DS15. I feel quite isolated or alone. |
| Stress and coping | Trapped | DS16. I feel trapped by what is happening to me. |

|  | | Supplementary Table S2: Items for Patient Dignity Inventory (PDI). | |  |
| --- | --- | --- | --- | --- |
| Dimensions | Node names | | Node numbers and items | |
| Symptom distress | Activity limitation | | PDI1. Not being able to carry out tasks associated with daily living. | |
| Symptom distress | Functional impairment | | PDI2. Not being able to attend to my bodily functions independently. | |
| Symptom distress | Physical distress | | PDI3. Experiencing physically distressing symptoms. | |
| Existential distress | Altered appearance | | PDI4. Feeling that how I look to others has changed significantly. | |
| Symptom distress | Depressed | | PDI5. Feeling depressed. | |
| Symptom distress | Anxious | | PDI6. Feeling anxious. | |
| Symptom distress | Illness uncertainty | | PDI7. Feeling uncertain about my illness and treatment. | |
| Symptom distress | Future concern | | PDI8. Worrying about my future. | |
| Symptom distress | Mental clouding | | PDI9. Not being able to think clearly. | |
| Dependency | Routine discontinuity | | PDI10. Not being able to continue with my usual routines. | |
| Existential distress | Personal transformation | | PDI11. Feeling like I am no longer who I was. | |
| Existential distress | Personal devaluation | | PDI12. Not feeling worthwhile or valued. | |
| Existential distress | Role incapacity | | PDI13. Not being able to carry out important roles. | |
| Existential distress | Life meaninglessness | | PDI14. Feeling that life no longer has meaning or purpose. | |
| Peace of mind | Contribution absence | | PDI15. Feeling that I have not made a meaningful and lasting contribution during my lifetime. | |
| Peace of mind | Unresolved matters | | PDI16. Feeling I have 'unfinished business' | |
| Peace of mind | Fearing spiritual emptiness | | PDI17. Concern that my spiritual life is not meaningful. | |
| Dependency | Self-perceived burden | | PDI18. Feeling that I am a burden to others. | |
| Dependency | Losing life control | | PDI19. Feeling that I don't have control over my life. | |
| Dependency | Privacy reduced | | PDI20. Feeling that my illness and care needs have reduced my privacy. | |
| Social support | Communal support deficiency | | PDI21. Not feeling supported by my community of friends and family. | |
| Social support | healthcare support deficiency | | PDI22. Not feeling supported by my health care providers. | |
| Dependency | Resilience depletion | | PDI23. Feeling like I am no longer able to mentally 'fight' the challenges of my illness. | |
| Dependency | Reality unaccepted | | PDI24. Not being able to accept the way things are. | |
| Social support | Respect deficit | | PDI25. Not being treated with respect or understanding by others. | |


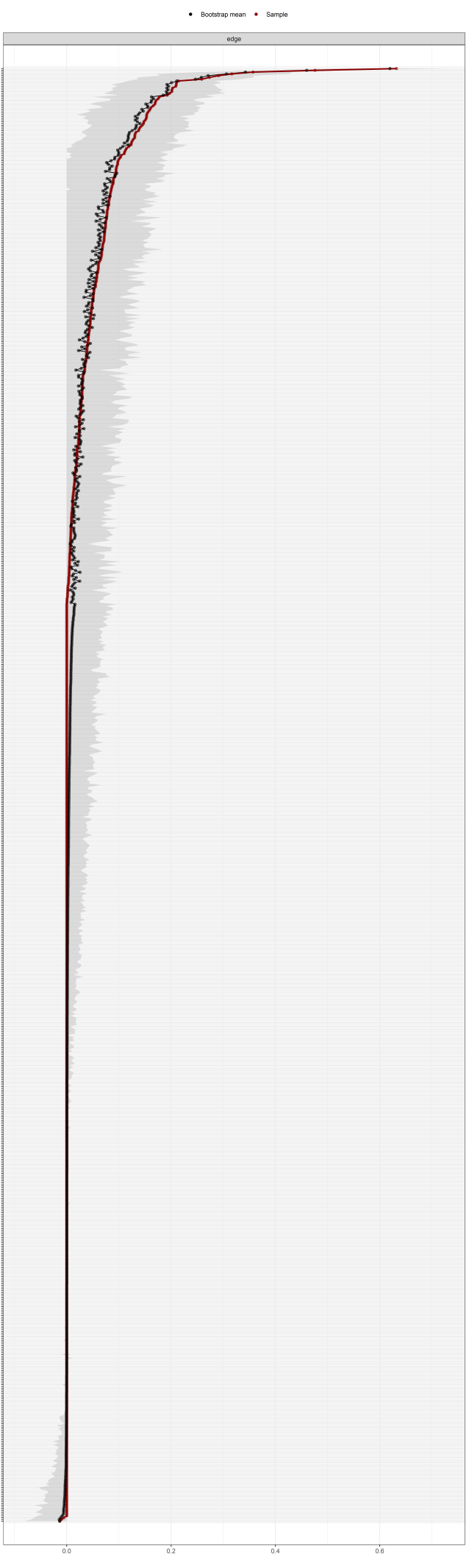


Supplementary Figure S1: The bootstrap 95% Confidence Intervals of edge weights for the bride network. (A higher-resolution figure with 600 dpi was provided separately)


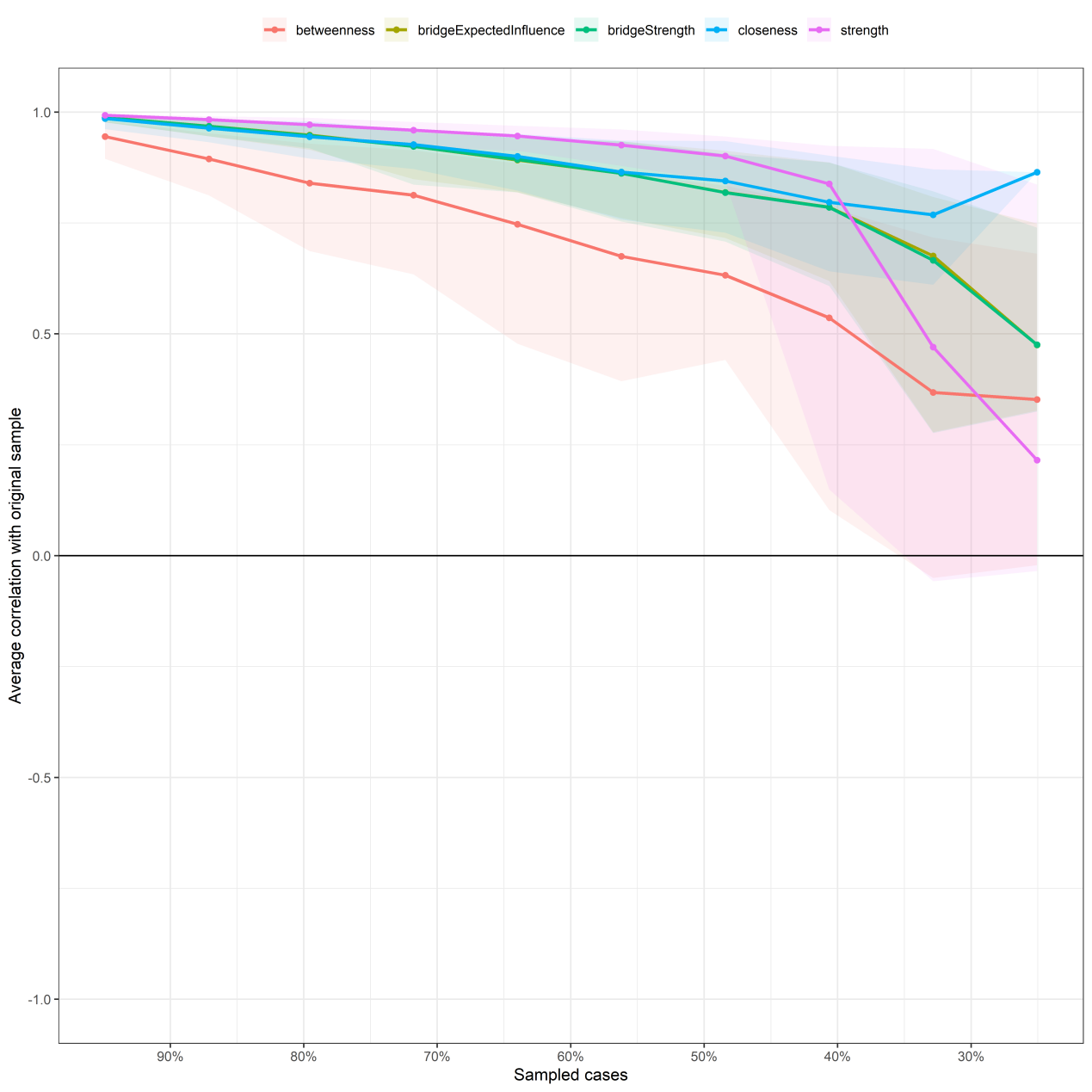


Supplementary Figure S2: The stability of centrality indices for nodes in bride network.

Supplementary Figure S3: Changes in sum score for alleviating and aggravating symptoms.


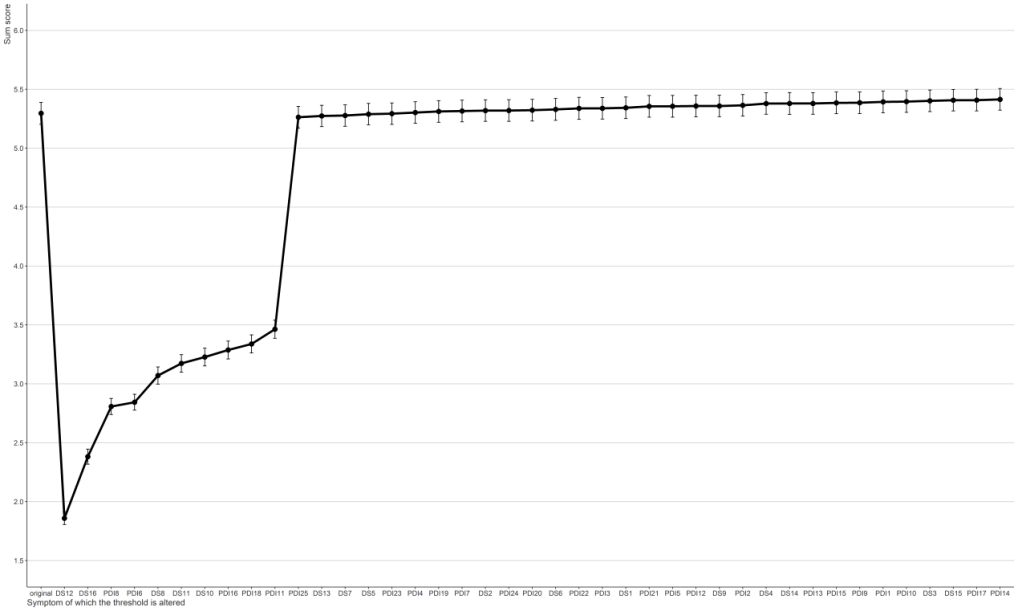


(A) alleviating symptom


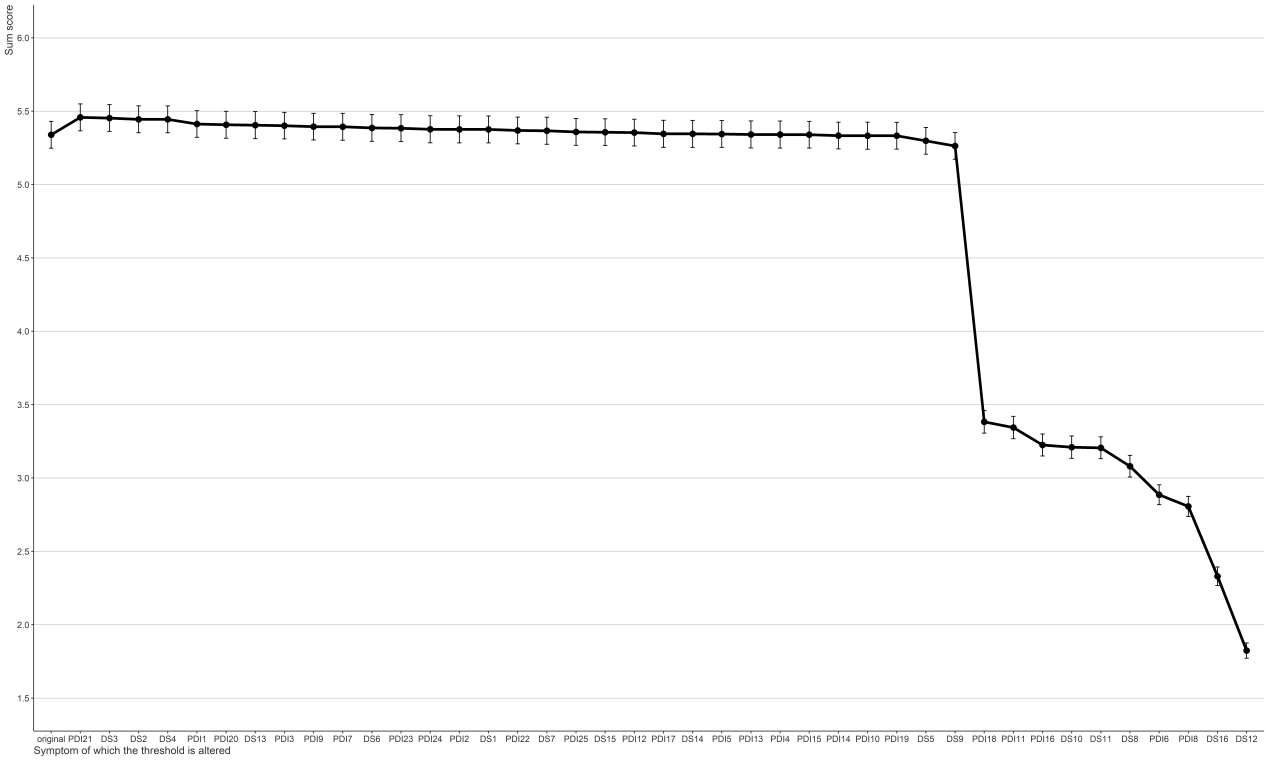


(B) aggravating symptom
